# Intestinal Perforation Secondary to Bortezomib‐Induced Autonomic Neuropathy

**DOI:** 10.1002/ccr3.70340

**Published:** 2025-04-01

**Authors:** Jose Antonio Sánchez Salas, Maria Jose Moreno Belmonte, Andrea Poveda García, Estela Ruiz Ruiz, Eva Soler Espejo, Valentin Cabanas Perianes, Ana María García Hernandez

**Affiliations:** ^1^ Department of Hematology Virgen de la Arrixaca University Hospital Murcia Spain

**Keywords:** Bortezomib, Intestinal perforation, Intestinal Pseudo‐obstruction, multiple myeloma, peripheral neuropathies

## Abstract

It is essential to evaluate both the patient's prior conditions and the severity of the current clinical presentation when deciding on BTZ toxicity management. It seems prudent to consider the permanent discontinuation of the drug in patients who have experienced at least grade 3 intestinal neuropathy and have structural abnormalities or other risk factors for intestinal perforation.

**Trial Registration:** NCT03710603

## Graphical abstract

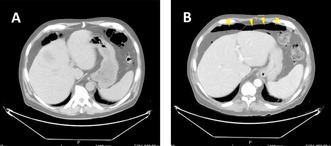

## 1 Introduction

Bortezomib (BTZ) is a proteasome inhibitor that prevents the breakdown of ubiquitinated proteins, causing their accumulation in the cytosol, which leads to cell cycle arrest and apoptosis. This drug was approved for the treatment of multiple myeloma (MM) in 2003 [1, 2], and since then, it has become part of the standard therapy for this disease, often in combination with other medications.

Among the adverse reactions associated with BTZ treatment are sensory neuropathy and autonomic nervous system (ANS) impairment, apparently caused by disruptions in axonal transport in microtubules, mitochondrial dysfunction, and oxidative stress, which result in neuronal dysfunction [2, 3].

Damage to the ANS is responsible for orthostatic hypotension and severe constipation with paralytic ileus experienced by some patients. The frequency of this latter complication varies depending on the study, with it being described in up to 12% of patients [4]. However, meta‐analyses reduce its incidence to 3.2% [5]. Only one case of toxic megacolon that ultimately led to intestinal perforation, possibly attributable to BTZ, has been reported [6].

We describe the case of a patient with MM without extramedullary disease who presented with paralytic ileus complicated by intestinal perforation in the context of therapy with daratumumab, BTZ, lenalidomide, and dexamethasone (D‐VRd). Here, we report the first case of BTZ‐induced intestinal perforation in a patient with a history of diverticulosis.

## 2 Case Presentation

### 2.1 Case Presentation and Investigation

A 60‐year‐old male with a history of hypertension, paroxysmal atrial fibrillation, gouty hyperuricemia, stage IIIb A3 chronic kidney disease due to MM‐related kidney disease, and colonic diverticulosis without a prior history of diverticulitis.

He was diagnosed with IgG Lambda Bence‐Jones positive MM (R‐ISS stage: 3, R‐ISS 3, and R2‐ISS 3.5 with high‐risk cytogenetics: delTP53, t (4;14), hypodiploidy [58% del16q23/MAF and monosomy 13]) without associated amyloidosis. He was considered a candidate for autologous peripheral hematopoietic stem cell transplantation (APHSCT). He thus began induction therapy with the quadruplet of D‐VRd based on the results of the PERSEUS [7]. The doses of the medications received are detailed in Table 1.

**TABLE 1:** D‐VRd doses are scheduled according to the cycle received.

Drug	Dose	Cycle			
		1	2	3	4
Daratumumab	1800 mg *sc*	+1 +8 +15 +22	+1 +8 +15	+1 +15	+1 +15
Bortezomib	1.3 mg/m^2^ *sc* (2.73 mg)	+1 +4 +8 +11	+1 +4 +8 +11	No	
	0.7 mg/m^2^ *sc* (1.45 mg)				+1 +4 +8 +11
Lenalidomide	5 mg^a^ *po*	+1 to +21	+1 to +21	+1 to +21	+1 to +15
Dexamethasone	40 mg *po*	+1 to +4+ and +9 to +12			
	20 mg *po*		+1 to +4 and +9 to +12	+1 to +4 and +9 to +12	+1 to +4 and +9 to +12

Abbreviations: *po*, oral; *sc*, subcutaneous.

^a^ Dose adjusted for renal function.

In the first cycle, the patient experienced a self‐limiting grade 1 constipation episode for which he did not seek medical consultation, and no treatment adjustment was necessary. The grading of neuropathy was performed according to the Common Terminology Criteria for Adverse Events (CTCAE) Version 5.0 [8], as the BTZ technical data sheet is based on these criteria [9].

**FIGURE 1:**
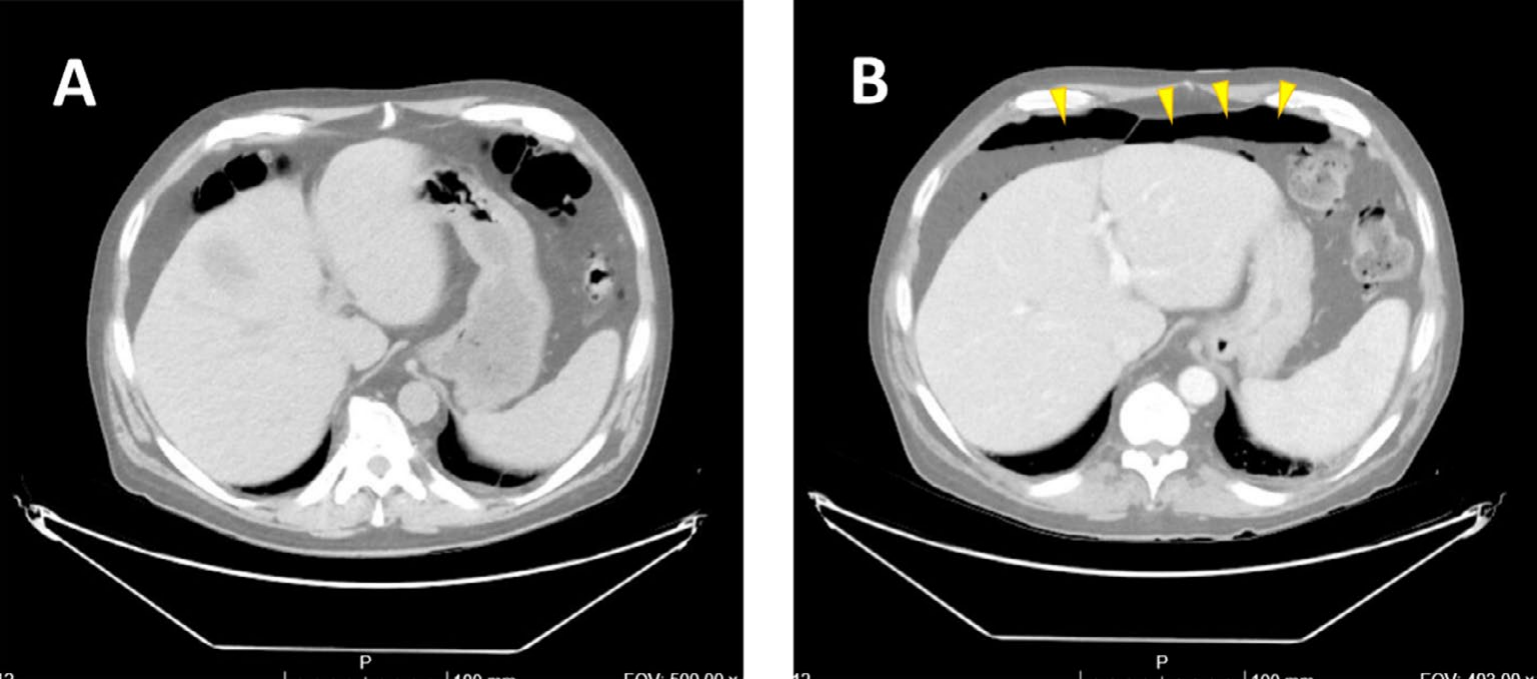
Comparison between urgent contrast‐enhanced abdominal CT scans performed during cycle 2 (A) and cycle 4 (B). The yellow arrows in B indicate free air in the abdomen, consistent with intestinal perforation.

On day +14 of the second cycle, the patient visited the emergency department, reporting grade 3 constipation and a subjective sensation of loss of gastrointestinal motility. He had received BTZ 3 days earlier. Physical examination revealed abdominal distension and mild tenderness on palpation without signs of peritoneal irritation. An abdominal CT scan (Figure 1A) showed multiple non‐inflamed diverticula in the colonic frame and distension of abdominal loops without signs of compromise. A nasogastric tube was placed, and prokinetic treatment with metoclopramide and erythromycin was initiated. After 3 days of hospitalization, the patient regained normal bowel movements and began tolerating oral intake, leading to his discharge.

Suspecting that the paralytic ileus was related to BTZ, the drug was not administered in the third treatment cycle. After the resolution of constipation, it was reintroduced in the fourth cycle with a reduced dose of 0.7 mg/m^2^ (1.45 mg).

**FIGURE 2:**
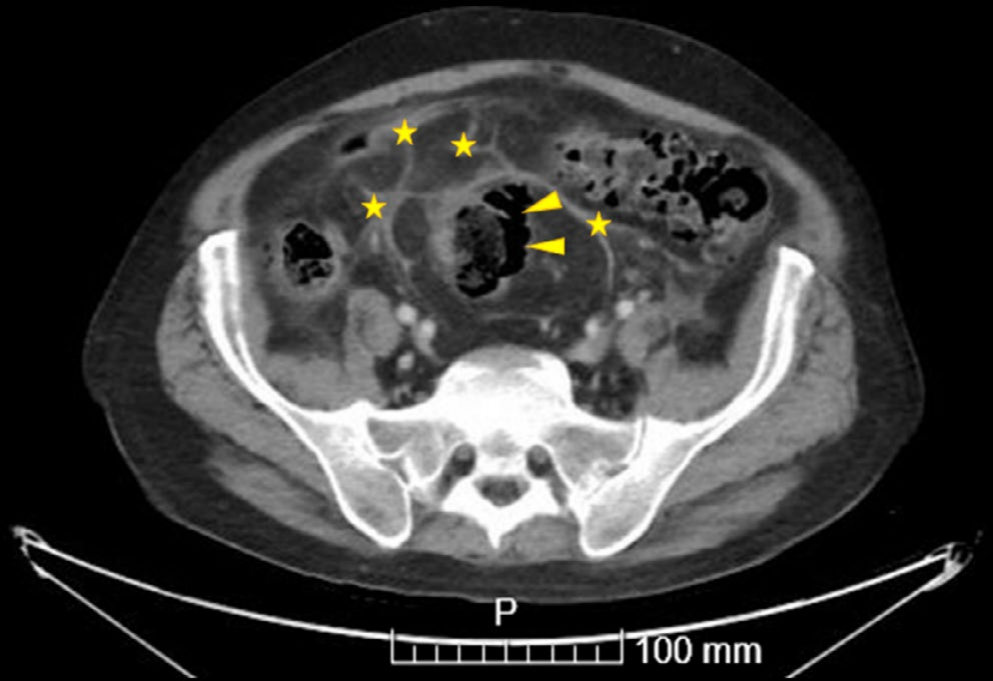
Abdominal CT scan performed during cycle 4. The yellow arrows indicate the discontinuity in the colonic wall at the sigmoid level. The yellow stars highlight the fat stranding suggestive of surrounding peritonitis.

On day +15 of the fourth cycle, the patient returned to the emergency department with the same symptoms. Upon examination, he presented generalized tenderness on palpation, abdominal guarding, and absent bowel sounds. An urgent abdominal CT scan (Figure 1B) was requested, revealing supra‐and inframesocolic pneumoperitoneum with parietal discontinuity in the sigmoid, attributable to perforation. These findings were associated with significant inflammatory changes, fat stranding, a small amount of partially collected free fluid, reactive colonic wall thickening, and probable focal peritonitis (Figure 2).

### 2.2 Treatment

Upon these findings, the patient was transferred to the operating room. During the emergency surgery, a large inflammatory phlegmon encompassing the sigmoid colon and loops of the small intestine was identified. A difficult release revealed a perforated diverticulum at the rectosigmoid junction. A resection of the affected area was performed, followed by a Hartmann's colostomy with a rectal stump. The decision to perform a Hartmann's procedure was preferred over primary repair since it involved a left‐sided colon perforation with significant inflammation and peritonitis [10].

### 2.3 Outcome and Follow‐Up

After hospitalization and recovery from surgery, two additional cycles of treatment were administered, but without BTZ, which was permanently discontinued. After six cycles, the patient achieved a stringent complete response and is now awaiting an autologous peripheral blood stem cell transplant.

## 3 Discussion

We suspect that the intestinal perforation was secondary to BTZ, as the patient had a longstanding diagnosis of diverticulosis without prior diverticulitis‐related complications. The symptoms preceding the perforation during the fourth cycle closely resembled those observed in the second cycle, both occurring after a comparable latency period. Notably, these symptoms were absent in the third cycle, when BTZ was not administered.

Bortezomib‐induced neuropathy may have led to intestinal obstruction and increased intraluminal pressure in the sigmoid colon. Combined with immunosuppression, this could have precipitated diverticulitis and, ultimately, sigmoid perforation.

In similar toxicity cases, the latency period for the onset of intestinal complications following BTZ administration varied [6, 11]; however, it generally fell within a timeframe of 2–3 weeks. Keating et al. [11] reported two cases, one of which involved paralytic ileus in a patient with kappa free‐light chain MM, occurring 14 days post‐treatment, closely resembling the case we describe.

Moreover, available reports suggest that toxicity does not necessarily manifest during the first cycle. Waller et al. [12] described a case of IgA MM in which the patient experienced a mild episode of intestinal ileus after the third cycle, managed on an outpatient basis, followed by a more severe episode requiring hospitalization during the fifth cycle. Similarly, Perfetti et al. [13] reported another case of IgA MM presenting with paralytic ileus after the second cycle.

Based on phase II and phase III clinical trials [14, 15], the BTZ technical data sheet recommends discontinuing the drug in cases of neuropathy with moderate symptoms that limit instrumental activities of daily living (grade 2) along with pain or severe symptoms that limit personal care activities (grade 3) [9].

However, in the absence of life‐threatening neuropathy (grade 4), the reintroduction of BTZ at a dose of 0.7 mg/m^2^ is recommended, as was decided in the fourth cycle for our patient.

The management strategies employed in the published cases were, however, heterogeneous. Focusing on grade 3 ileus: Waller et al. opted for permanent discontinuation of BTZ [12], Mele et al. [16] initially discontinued BTZ but later reintroduced it at 25% of the initial dose, and Keating et al. reduced the initial dose from 1.3 mg/m^2^ to 1.0 mg/m^2^ in both cases [11].

In the case reported by Bhalla et al. [6] which involved an ileus of similar severity to that of our patient (grade 4 with associated intestinal perforation), BTZ was permanently discontinued.

Keating et al. suggest that to reduce the risk of paralytic ileus in patients with comorbidities such as renal insufficiency with glomerular filtration < 30 mg/mL, the BTZ dose should be reduced to 1 mg/m^2^ from the beginning [11]. In parallel, Sousa‐Amorim et al. reported two cases of patients without MM who received BTZ as a treatment for antibody‐mediated rejection in kidney transplantation. In these cases, intestinal toxicity appeared earlier (during the first cycle), leading to the permanent discontinuation of the drug [17]. On several occasions, our patient had fluctuating glomerular filtration, which had been below 30 mg/mL.

We further propose that BTZ be definitively discontinued in patients who have already presented with neuropathy in the form of grade 3 constipation or intestinal ileus requiring hospitalization, especially those with pre‐existing risk factors for perforation, such as intestinal diverticulosis, which could potentially evolve into diverticulitis due to abdominal dysmotility.

## 4 Conclusion

Management of complications associated with BTZ should consider the patient's comorbidities and prior status. In cases of gastrointestinal neuropathy, it may be prudent to discontinue the drug if the patient develops severe neuropathy (grade 3 or higher) and presents additional conditions that could contribute to serious complications, such as intestinal perforation, with an individualized approach for each case.

## Author Contributions

**Jose Antonio Sánchez Salas:** writing – original draft. **Maria Jose Moreno Belmonte:** methodology, supervision. **Andrea Poveda García:** writing – original draft. **Estela Ruiz Ruiz:** data curation, investigation. **Eva Soler Espejo:** conceptualization, resources. **Valentin Cabanas Perianes:** supervision, writing – review and editing. **Ana María García Hernandez:** supervision, validation, visualization, writing – review and editing.

## Consent

Written informed consent was obtained from the patient to publish this report in accordance with the journal's patient consent policy.

## Conflicts of Interest

The authors declare no conflicts of interest.

## Supporting information


AppendixS1.

## Data Availability

Data sharing not applicable to this article as no datasets were generated or analysed during the current study.

## References

[1] R. C. Kane , P. F. Bross , A. T. Farrell , and R. Pazdur , “Velcade: U.S. FDA Approval for the Treatment of Multiple Myeloma Progressing on Prior Therapy,” Oncologist 8, no. 6 (2003): 508–513, 10.1634/theoncologist.8-6-508. 14657528

[2] S. Yamamoto and N. Egashira , “Pathological Mechanisms of Bortezomib‐Induced Peripheral Neuropathy,” International Journal of Molecular Sciences 22, no. 2 (2021): 888, 10.3390/ijms22020888. 33477371 PMC7830235

[3] W. Yan , Z. Wu , Y. Zhang , et al., “The Molecular and Cellular Insight Into the Toxicology of Bortezomib‐Induced Peripheral Neuropathy,” Biomedicine & Pharmacotherapy 142 (2021): 112068, 10.1016/j.biopha.2021.112068. 34463262

[4] A. A. Argyriou , G. Cavaletti , J. Bruna , A. P. Kyritsis , and H. P. Kalofonos , “Bortezomib‐Induced Peripheral Neurotoxicity: An Update,” Archives of Toxicology 88, no. 9 (2014): 1669–1679, 10.1007/s00204-014-1316-5. 25069804

[5] K. Scott , P. J. Hayden , A. Will , K. Wheatley , and I. Coyne , “Bortezomib for the Treatment of Multiple Myeloma,” Cochrane Database of Systematic Reviews 4, no. 4 (2016): CD010816, 10.1002/14651858.CD010816.pub2. 27096326 PMC10387344

[6] P. Gill , V. Bhalla , V. Mulkareddy , and K. Sitaraman , “Toxic Megacolon Leading to Bowel Perforation: A Rare Adverse Effect of Bortezomib,” Journal of Clinical Images and Medical Case Reports 4, no. 3 (2023): 2333, 10.52768/2766-7820/2333.

[7] European Myeloma Network BV , “A Phase 3 Study Comparing Daratumumab, Velcade (Bortezomib), Lenalidomide, and Dexamethasone (D‐VRd) vs. Velcade, Lenalidomide, and Dexamethasone (VRd) in Subjects With Previously Untreated Multiple Myeloma Who Are Eligible for High‐Dose Therapy,” ClinicalTrials.gov, Report No.: NCT03710603, https://clinicaltrials.gov/study/NCT03710603.

[8] U.S. Department of Health and Human Services, National Cancer Institute , “Common Terminology Criteria for Adverse Events (CTCAE) Version 5.0,” (2017), https://ctep.cancer.gov/protocolDevelopment/electronic_applications/docs/CTCAE_v5_Quick_Reference_8.5x11.pdf.

[9] European Medicines Agency , Velcade: EPAR – Product Information (European Medicines Agency, 2009), https://www.ema.europa.eu/en/medicines/human/EPAR/velcade.

[10] M. Pisano , L. Zorcolo , C. Merli , et al., “2017 WSES Guidelines on Colon and Rectal Cancer Emergencies: Obstruction and Perforation,” World Journal of Emergency Surgery: WJES 13 (2018): 36, 10.1186/s13017-018-0192-3. 30123315 PMC6090779

[11] M. Keating and C. A. Dasanu , “Strategy to Reduce Bortezomib‐Induced Paralytic Ileus in Patients With Myeloma and Impaired Renal Function,” BMJ Case Reports 2016 (2016): 217000, 10.1136/bcr-2016-217000. PMC5174818 27899388

[12] J. M. Waller , J. C. Moretto , and K. B. Knopf , “Multiple Significant Bortezomib‐Related Toxicities in One Patient: Case Report and Literature Review,” Clinical Lymphoma & Myeloma 9, no. 3 (2009): E1–E4, 10.3816/CLM.2009.n.051. 19525183

[13] V. Perfetti , G. Palladini , L. Brunetti , et al., “Bortezomib‐Induced Paralytic Ileus Is a Potential Gastrointestinal Side Effect of This First‐in‐Class Anticancer Proteasome Inhibitor,” European Journal of Gastroenterology & Hepatology 19, no. 7 (2007): 599–601, 10.1097/MEG.0b013e32811ebffe. 17556909

[14] P. G. Richardson , B. Barlogie , J. Berenson , et al., “A Phase 2 Study of Bortezomib in Relapsed, Refractory Myeloma,” New England Journal of Medicine 348, no. 26 (2003): 2609–2617, 10.1056/NEJMoa030288. 12826635

[15] J. F. San‐Miguel , P. G. Richardson , P. Sonneveld , et al., “Efficacy and Safety of Bortezomib in Patients With Renal Impairment: Results From the APEX Phase 3 Study,” Leukemia 22, no. 4 (2008): 842–849, 10.1038/sj.leu.2405087. 18200040

[16] G. Mele , M. R. Coppi , A. Melpignano , and G. Quarta , “Paralytic Ileus Following “Subcutaneous Bortezomib” Therapy: Focus on the Clinical Emergency‐Report of Two Cases,” Clinical and Experimental Medicine 16, no. 1 (2016): 99–101, 10.1007/s10238-015-0337-6. 25600700

[17] E. De Sousa‐Amorim , I. Revuelta , F. Diekmann , et al., “High Incidence of Paralytic Ileus After Bortezomib Treatment of Antibody‐Mediated Rejection in Kidney Transplant Recipients: Report of 2 Cases,” Transplantation 99, no. 11 (2015): e170–e171, 10.1097/TP.0000000000000930. 26492053

