# Supplementary material for: Intestinal Perforation Secondary to Bortezomib‐Induced Autonomic Neuropathy
Source: Clin Case Rep. 2025 Apr 1;13(4):e70340. doi: 10.1002/ccr3.70340 (PMC11959410; doi:10.1002/ccr3.70340)
Supplement: Supplementary file 1 — AppendixS1. [file CCR3-13-e70340-s001.rtf]

Figure 1. Comparison between urgent contrast-enhanced abdominal CT scans performed during cycle 2 (A) and cycle 4 (B). The yellow arrows in B indicate free air in the abdomen, consistent with intestinal perforation.

Figure 2. Abdominal CT scan performed during cycle 4. The yellow arrows indicate the discontinuity in the colonic wall at the sigmoid level. The yellow stars highlight the fat stranding suggestive of surrounding peritonitis.

Table 1. D-VRd doses scheduled according to the cycle received. sc: subcutaneous, po: oral. *Dose adjusted for renal function.
